# Beyond insulin: rethinking cellular metabolism

**DOI:** 10.1186/1753-6561-6-S3-O25

**Published:** 2012-06-01

**Authors:** Craig B Thompson

**Affiliations:** 1 Memorial Sloan-Kettering Cancer Center, New York, NY 10065, USA

## 

The latest cancer therapeutics are almost all inhibitors of oncogenes or their downstream mediators. With the exception of imatinib, most such drugs have been disappointing in the clinic. In contrast, existing successful cancer drugs have a therapeutic index because they render the consequences of oncogene activation selectively toxic to the cancer cell. A renewed focus on this type of approach appears warranted and should be considered.

